# Exogenous Applications of Bio-fabricated Silver Nanoparticles to Improve Biochemical, Antioxidant, Fatty Acid and Secondary Metabolite Contents of Sunflower

**DOI:** 10.3390/nano11071750

**Published:** 2021-07-03

**Authors:** Syeda Umber Batool, Bilal Javed, Sohail, Syeda Sadaf Zehra, Zia-ur-Rehman Mashwani, Naveed Iqbal Raja, Tariq Khan, Haifa Abdulaziz Sakit ALHaithloul, Suliman Mohammed Alghanem, Amina A. M. Al-Mushhin, Mohamed Hashem, Saad Alamri

**Affiliations:** 1 Department of Chemical and Life Sciences, Qurtuba University of Science & Information Technology, Peshawar 25000, Khyber Pakhtunkhwa, Pakistan; syedaumberbatool5@gmail.com; 2 Department of Botany, PMAS Arid Agriculture University, Rawalpindi 46300, Punjab, Pakistan; drnaveodraja@gmail.com; 3 Institute of Biology/Plant Physiology, Humboldt-University Zü Berlin, 10115 Berlin, Germany; sohail.botanist@hotmail.com; 4 Department of Botany, The Islamia University of Bahawalpur, Bahawalpur 63100, Punjab, Pakistan; sadaf.zahra@iub.edu.pk; 5 Department of Biotechnology, University of Malakand, Chakdara, Lower Dir 18800, Khyber Pakhtunkhwa, Pakistan; tariqkhan@uom.edu.pk; 6 Biology Department, College of Science, Jouf University, Sakaka 2014, Saudi Arabia; haifasakit@ju.edu.sa; 7 Biology Department, Faculty of Science, Tabuk University, Tabuk 71491, Saudi Arabia; s-alghanem@ut.edu.sa; 8 Department of Biology, College of Sciences and Humanities in AlKharj, Prince Sattam Bin Abdulaziz University, AlKharj 16278, Saudi Arabia; a.almushhin@psau.edu.sa; 9 Department of Biology, College of Science, King Khalid University, Abha 61413, Saudi Arabia; drmhashem69@yahoo.com (M.H.); amri555@yahoo.com (S.A.); 10 Botany and Microbiology Department, Faculty of Science, Assiut University, Assiut 71516, Egypt

**Keywords:** silver nanoparticles, agro-morphological parameters, enzymatic activities, biochemical analysis, secondary metabolites

## Abstract

The present study involved the bio-fabrication of silver nanoparticles (AgNPs) by using the *Euphorbia helioscopia* L. leaves aqueous extract to improve the production of secondary metabolites in industrially important sunflower (*Helianthus annuus* L.) plants. Phyto-fabrication of AgNPs was confirmed by using spectrophotometry, SEM imaging and X-ray diffraction analysis. The morphological and optical characterization manifested that the AgNPs are crystalline and exist in the size range of 30–100 nm. Various concentrations (10, 20, 40, 60, 80 and 100 mg/L) of AgNPs were applied in combinations on sunflower seeds and crop plants. The effects of biosynthesized AgNPs were evaluated for agro-morphological parameters (plant height, flowering initiation and seed weight), biochemical metabolites (chlorophyll, proline, soluble sugar, amino acid and protein contents) and enzymatic activities (superoxide dismutase and ascorbate peroxidase) in sunflower and 60 mg/L concentration of AgNPs on sunflower seeds and foliar sprays on plants in combination were found to be effective to elicit biochemical modifications to improve secondary metabolites. It was also observed experimentally that 60 mg/L concentration of AgNPs improved the biochemical, fatty acid and enzymatic attributes of sunflower plants, which in turn improved the plant agro-morphological parameters. Near-infrared spectroscopic analysis results confirmed the improvement in the seed quality, oil contents and fatty acid composition (palmitic acid, oleic acid and linoleic acid) after the applications of AgNPs. The findings of the present investigation confirm the exogenous applications of bio-fabricated AgNPs in combinations on seeds and plants to improve the plant yield, seed quality and secondary metabolite contents of the sunflower plants.

## 1. Introduction

The demand for food is increasing with every passing year because of the drastic increase in the world’s population. It is estimated that the world’s population will reach 9.2 billion people at the end of 2050 [1,2,3]. This rapid increase in the population will require a 70% increase in the world’s agricultural production to meet the growing demand for food, compared with today’s levels. To ensure food security, there must be a rapid increase in the improvement and the production of crop plants [4,5].

Nanotechnology has a huge potential to improve agricultural practices and crop production. It has been shown that different nanomaterials have been applied to the plants to increase the production of plants’ biomass and yield. Tremendous applications of nanotechnology attracted the scientific community to take special initiatives to promote the agriculture industry [6,7]. Metal nanoparticles, nonmetal materials, carbon nanotubes and magnetic particles are various types of nanomaterials that are employed for the improvement in the physiological and biochemical properties of important crop plants [8,9]. Among these nanomaterials, silver nanoparticles (AgNPs) have been used extensively to improve agricultural and industrial products for the past few decades. AgNPs have the potential to trigger the growth of plants; enhance secondary metabolism to produce higher amounts of phenolics and flavonoids, fatty acids and lipids; and enhance other morphological parameters, such as shoot length, flowering and seed yield. Looking at the immense need to bridge the gaps related to sunflower yield and the important features of AgNPs, this study was designed to investigate the effects of AgNPs on the growth, seed quality, oil yield and secondary metabolism of the sunflower plant [2,10].

*Helianthus annuus* (sunflower) is one of the important food plants that belongs to the family *Asteraceae* and plays a very important role in meeting the food demands and fulfilling the nutritional requirements [7]. *H. annuus* is mainly harvested as an oilseed crop that yields oil in the proportion which is 40–50% of the total dry mass of the plant. *H. annuus* was introduced in Pakistan ca. 40–50 years back by the Pakistan Agriculture Research Council (PARC) because the environmental conditions of the country are favorable for the cultivation and it can be grown twice a year to fulfill the demand for vegetable oil in Pakistan. Sunflower oil is rich in important fatty acids such as linoleic acid (55–70%) and oleic acid (20–25%). Oleic acid has monosaturated omega-9 fatty acids, which help to reduce the risk of heart attack by converting the low-density lipoproteins to high-density lipoproteins [11,12]. Oleic acid also helps in oil stability, reduces oxidative stress and works as a preservative. The sunflower plants have higher contents of poly-saturated fatty acids (31%) as compare to sesame (25.5%), flax (22.4%), cottonseed (18.1%), peanut (13.1%) and soybean (3.5%) [13,14].

Furthermore, due to the presence of phytochemicals such as phenolic compounds, antioxidants, poly-saturated fatty acids, the sunflower is also considered an important medicinal plant and has nutraceutical value. Its seeds are used for the treatment of different diseases such as chest infection, cold, heart diseases, chronic pulmonary infection and pertussis [15,16,17]. Oleic acid suppresses the effect of the Her-2/neu gene which involve in the formation of breast cancer [18].

However, the acreage and production are always varying due to various production constraints such as climate and economic constraint. This ultimately fails to cope with the increasing demand. For instance, the production was 338,000 tons compared to the demand for this oilseed crop that was 1,262,000 tons during the year [5,19,20]. The above review provides the baseline information to use nanotechnological tools to improve productivity and enhance the contents of the secondary metabolites of the sunflower plants. Herein we report the exogenous applications of AgNPs on *Helianthus annuus* seeds and plants to improve the quality of the seed, oil contents, fatty acids, agro-morphological parameters and the secondary metabolites.

## 2. Materials and Methods

### 2.1. Biogenesis of AgNPs by Using Aqueous Extract of Euphorbia Helioscopia

The young leaves of *Euphorbia helioscopia* were used for the biosynthesis and the stabilization of AgNPs. Fresh plant leaves of *E. heliopscopia* were collected from wild plants growing freely in the gardens of PMAS-Arid Agriculture University, Rawalpindi Pakistan (33°38′52.9″ N 73°04′52.1″ E). For this purpose, 10 g of *E. helioscopia* leaves were washed with the distilled water to remove the dust particles and then dried at room temperature. After that, the leaves were crushed and mixed with 100 mL of distilled water. The plant aqueous extract was prepared by heating the plant material in water for 20–25 min at 45 °C. The plant aqueous extract was filtered thrice to remove debris and then refrigerated at 4 °C for further use. Then 2 mg of AgNO_3_ was added in 500 mL of distilled water (23.55 µM). The 10 mL of plant aqueous extract was mixed with 500 mL of AgNO_3_ and agitated by using a magnetic stirrer until the color of the reaction mixture turned dark brown. The synthesis of the AgNPs was confirmed by measuring the absorbance of the colloidal mixture by using a UV–Visible spectrophotometer (Beckman DU-640, Missouri, TX, USA) in the range of 200 to 800 nm of the light wavelength [21].

### 2.2. Morphological and Optical Characterization of AgNPs

Different analytical characterization techniques were used to confirm the synthesis of AgNPs, as well as to determine their size and crystal structure. The UV–visible spectrophotometry (Beckman DU-640) was used to confirm the synthesis of AgNPs. The absorbance of the light in the reaction mixture was recorded in the range of 200 to 800 nm of the light wavelength [22].

The scanning electron microscope (SEM) (JSM-7610F Plus, Tokoy, Japan) was used to determine the size and morphology of AgNPs. The sample was prepared by simply dropping the colloidal suspension of AgNPs on a carbon-coated copper grid. The residues were removed by using the blotting paper, and the sample was left to dry for 5 min, under a mercury lamp [23].

The crystalline nature of the AgNPs was confirmed by using the X-ray diffraction (XRD) (Bruker D2 Phaser, Coventry, UK) analysis with the monochromatic Cu-Kα1 radiation at a 2θ angle between 30° and 80° [24].

### 2.3. Treatment of Sunflower Seeds with AgNPs

The biosynthesized AgNPs were applied exogenously on the seeds and the plant body of *H. annuus* for in vitro analysis. The plants were grown in the greenhouse to study the effects of phyto-synthesized AgNPs on different physiological and biochemical aspects of the sunflower. Three sets of experiments were arranged that were having further treatments: (i) seeds treatment with various concentrations of AgNPs, (ii) foliar spray of AgNPs on plant body, and (iii) both seed treatment and foliar spray. For this purpose, the seeds of a specific variety (PARSUM-3) were obtained from the National Agriculture Research Center of Pakistan. The seeds were soaked in different concentrations (10, 20, 40, 60, 80 and 100 mg/L) of the AgNPs, while the control seeds were treated with the distilled water. Three seeds were used for each treatment and were soaked in 10 mL of colloidal AgNPs suspension for 24 h. The colloidal AgNPs solution was sonicated for a few minutes before their application on sunflower plants and seeds. The plants were harvested after ca. 60 days from each pot to examine the agronomical, morphological, biochemical and enzymatic effects of AgNPs on the sunflower plant. In the next experimental treatment, the first foliar spray was applied on plants at the flowering stage after 30 days of the seeding stage, while the second foliar spray was applied on sunflower plants when the flowers were fully emerged and grown with the gap of 30 days after the first foliar spray. Each plant with a different treatment received a different concentration of AgNPs. AgNPs were diluted to 500 mL of distilled water and were used as a foliar spray.

In the next experimental setup, both foliar sprays and seed treatments were given to the plants simultaneously to study their synergistic effects. The complete treatment layout is given in Table 1.

### 2.4. Agro-Morphological Evaluation of Plants Treated with AgNPs

The plants treated with AgNPs were collected after ca. 60 days of treatment and analyzed for the change in agro-morphological parameters, including initiation of flowering, shoot length, seed weight, number of filled seeds, number and weight of unfilled seeds, and flower-head diameter. Experiments were performed in a triplicate manner.

For the determination of flowering initiation, the number of days the plant took till the production of early flowers was noted for each treatment. The count was made based on 50% of flowering days. The following growing degree days (GDD) formula was used for the analysis:

$$GDD=\left[\frac{\left(Maximum\;Temperature-Minimum\;Temperature\right)}{2}\right]-Base\;Temperature$$

where the Max Temp and Min Temp stand for the average of the daily maximum and minimum temperatures, respectively.

Furthermore, the height of the plant was measured as the shoot length of the plant by measuring the average length from the surface to the tip of the plant in centimeters (cm). Similarly, seeds were harvested for each replicate and dried at a temperature of 30 to 40 °C. A hundred seeds were counted and weighed (gram) by an analytical balance. The volume weight of seeds per 100 milliliters was also measured. Further, the number of seeds filled inside, as well as those unfilled, was counted. The head diameter of the sunflower from each replicate was measured by using the ruler in centimeters (cm).

### 2.5. Antioxidant Enzymatic Activity Measurement of the Plants Treated with AgNPs

#### 2.5.1. Superoxide Dismutase (SOD) Assay

A total of 0.5 g of the sunflower leaves was crushed in 10 mL of phosphate buffer (50 mM, pH 7.8) and centrifuged at 6000 rpm. Then 0.1 mL of the supernatant was taken in the test tube and added with 13 mM of methionine (0.8 mL), 75 M of nitro blue tetrazolium (0.5 mL), 0.1 mM of EDTA (0.7 mL) and 2 M of riboflavin (1 mL) to make the total volume of 3 mL. This reaction mixture was placed under a fluorescent lamp (30 W) for 30 min and then in the dark for 10 min. The absorbance of the sample was recorded at 560 nm by using the spectrophotometer (Beckman DU-640, USA). The activity was expressed as a unit per minute per mg of protein. The following formula was used for the quantification of the SOD activity [25]:

$$SOD=\frac{\mathrm{Absorbance}\;\mathrm{of}\;\mathrm{Sample}\;\text{×}\;k\;\mathrm{Value}\;\text{×}\;\mathrm{Dilution}\;Factor}{Weight\;\mathrm{of}\;\mathrm{Sample}}$$

#### 2.5.2. Ascorbate Peroxidase (APx) Assay

A total of 0.5 g of the crushed sunflower leaves was mixed with 50 mM of potassium phosphate (pH 7) (5 mL) and centrifuged at 6,000 rpm to collect the supernatant. Then 0.1 mL of the supernatant was taken and added with 0.5 mM of ascorbate (0.5 mL), 0.1 mM of hydrogen peroxide (0.2 mL) and 0.1 mM of EDTA (0.3 mL) to make the total volume of 1 mL. The absorbance of the reaction mixture was recorded at 290 nm for 10 to 30 s after adding hydrogen peroxide. The results were expressed as unit per minute per mg of protein. The APx activity was calculated according to the following formula [26]:

$$Enzyme\;Activity=\frac{\Delta Abs\times Total\;Assay\;Volume}{\Delta t\times \varepsilon \times l\times Enzyme\;Sample\;Volume}$$

where absorbance coefficient (ε) = 2.8 mM^−1^ cm^−1^, and the diameter of the cuvette = 1 cm.

### 2.6. Biochemical Parameters of the Plants Treated with AgNPs

#### 2.6.1. Determination of the Chlorophyll Contents

The Chlorophyll content of the plants treated with AgNPs was determined according to the protocol of Reference [27]. A total of 0.5 g of leaves were washed with the distilled water and crushed in 5 mL of ethanol. The clean sand was added to facilitate the extraction process. The extract was filtered, and the absorbance was measured at 663, 645 and 652 nm for chlorophyll *a*, chlorophyll *b* and total chlorophyll content determination.

#### 2.6.2. Determination of Proline Contents

To determine the quantity of the proline, the protocol of Reference [28] was followed. A total of 0.5 g of the plant material was crushed in 10 mL of 3% sulfosalicylic acid and the mixture was filtered twice by Whatman filter paper. After this, an equal volume (2 mL) of filtrate, acid ninhydrin and glacial acidic acid were mixed and placed in a water bath for one hour at 100 °C. The test tubes with samples were placed in an ice bath to quench the reaction, and 4 mL of toluene was added for the extraction of the reaction mixture, followed by shaking the test tube for 15 to 20 s and then cooling at room temperature. The absorbance of the reaction mixture was recorded at 520 nm of the light wavelength, and the proline content was calculated according to the following formula:

Sample Absorbance×Dilution Factor×k Value/ Fresh Weight of the Plant

#### 2.6.3. Determination of Soluble Sugar Contents

For the quantification of the soluble sugar in the sunflower plants treated with AgNPs, 0.5 gm of leaves were crushed into 90% of ethanol and distilled water was added to make the total volume at 25 mL. In the next step, 1 mL of 5% phenol and 5 mL of concentrated sulfuric acid were added to the supernatant. The absorbance of the reaction mixture was measured at 485 nm of the light wavelength, and the quantity of soluble sugar was calculated by using the following formula [29]:

Sample Absorbance×Dilution Factor×k Value/ Fresh Weight of the Plant

#### 2.6.4. Determination of Free Amino Acid Contents

The protocol of Reference [30] was followed for the determination of free amino acid content (FMA). A total of 0.2 gm of the plant extract was crushed in 10 mL of phosphate buffer. The reaction mixture was prepared by adding 1 mL of supernatant, 1 mL of 10% pyridine and 1 mL of 2% ninhydrin solution. The absorbance was recorded at 570 nm of the light wavelength.

#### 2.6.5. Determination of Total Protein Contents

For the determination of the total protein content, the protocol of Reference [1] was followed. A total of 0.5 g of the leaves were crushed in 10 mL of phosphate buffer and centrifuged, followed by the addition of 3 mL of Bradford reagent (five times diluted) in 0.5 mL of supernatant. The absorbance of the reaction mixture was measured at 595 nm of the light wavelength, and the total protein content was determined according to the following formula:

Sample Absorbance×Dilution Factor×k Value/ Fresh Weight of the Plant

### 2.7. Determination of the Oil Content and Fatty Acid Composition of the Plants Treated with AgNPs

For the determination of biochemical effects of AgNPs on seed quality, the oil content and fatty acid composition were analyzed through near-infrared spectroscopic (NIRS) analysis (6500, Foss NIR Systems Inc., Silver Spring, MD, USA). One hundred seeds from each treatment were collected for the determination of seed oil and fatty acid contents. For the analysis, the husk of the seeds was removed by a knife and then crushed. Ca. 5 mL of diethyl ether was added to the 1 g of crushed seeds in a small container, by shaking at regular intervals for five hours. In this way, the solvent was evaporated, and the oil was extracted. By using the NIRS scanning, the reflectance spectra were taken at the wavelength of 400 to 2,500 nm, at an interval of 2 nm. The oil was scanned by adding 2 or 3 drops of oil on the glass fiber disk. The oil content and fatty acids, such as oleic acid, linoleic acid and stearic acid, were measured from the NIRS calibration equation, and the spectra were taken between 1,100 and 2,500 nm (Perez et al., 1998). The oil contents were determined by nuclear magnetic resonance, and fatty acid composition was determined by gas-liquid chromatography [11,13].

### 2.8. Data Analysis

The experiments were performed in triplicate. The standard deviation and the mean of the results were used to draw graphs and charts. Statistix^®^ 8.1 was used for the statistical analysis. The analysis of the variance (ANOVA) was used to determine the statistical significance of the results, and *p* < 0.05 was considered a significant difference. Duncan’s multiple range test was used to determine the significance of the difference among treatment groups.

## 3. Results and Discussion

### 3.1. Phyto-Synthesis of AgNPs

**Figure 1:**
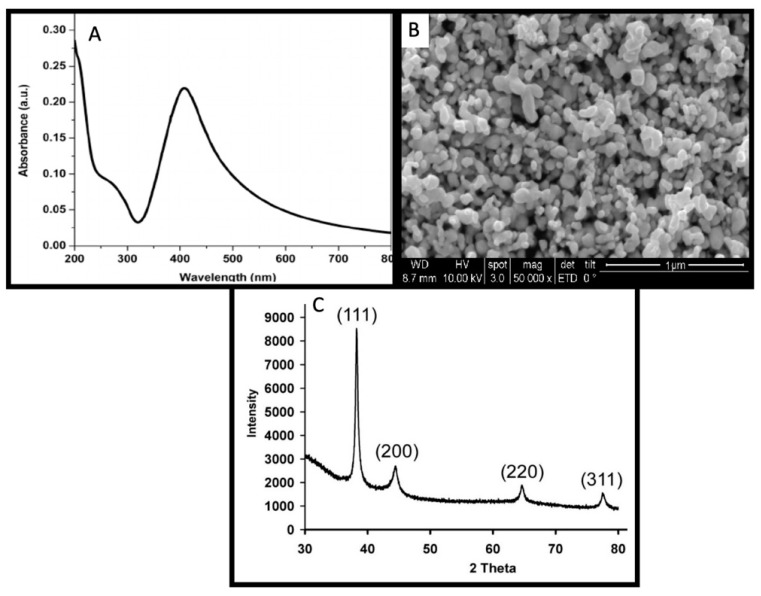
Morphological and optical characterization of bio-fabricated AgNPs: (**A**) UV–visible spectrum, (**B**) SEM image analysis and (**C**) XRD analysis.

Phyto-synthesis of the AgNPs has advantages over routine physical and chemical methods of synthesis. The use of the reducing and stabilizing abilities of the plant secondary metabolites result in the synthesis of biocompatible nanoparticles which have potential functional groups that originate from the plant secondary metabolites to reduce the toxic effects of nanomaterials. Plant secondary metabolites also function as a catalyst and play a contributing role to accelerate the rate of reduction of bulk Ag into Ag^0^ [3,31]. Herein we report the green synthesis of AgNPs by using the reducing, stabilizing and catalyzing abilities of *Euphorbia helioscopia* leaves aqueous extract. Change in the color of the reaction mixture from light brown to dark brown or tea color was considered as an initial sign to report synthesis. The synthesis was confirmed by observing a characteristic surface plasmon resonance (SPR) band in between 200 and 800 nm of the light wavelength, while the highest peak was observed at 420 nm to confirm the synthesis (Figure 1A). The SPR band is a response of the oscillating electromagnetic light waves in coordination with the vibrating nanoparticles in the colloidal suspension [32]. The yield percentage of the AgNPs synthesis reaction was measured (85%) by dividing the collective mass of the synthesized AgNPs by the mass of Ag used to initiate the synthesis reaction. In the latter experiments, the bio-fabricated nanoparticles were applied exogenously on the *Helianthus annuus* plants and seeds to enhance the secondary metabolites, oil contents and fatty acids of the economically important sunflower plants and to comprehensively study the effects of the AgNPs on morphological/agronomical, physiological and enzymatic/biochemical parameters.

### 3.2. Physical and Optical Characterization of AgNPs

The structural and optical characterization of AgNPs was performed by using various analytical characterization techniques such as SEM and XRD analysis. SEM analysis showed a detailed 3D image of the AgNPs surface with a high magnification of 50,000 at 10 kV. The SEM images revealed that the nanoparticles are spherical and anisotropic while some nanoparticles were observed irregular in shape (Figure 1B). The SEM images also confirmed that the nanoparticles are less than 100 nm in the size range.

The crystalline nature of the AgNPs was confirmed by performing the XRD analysis of the powdered sample. The peak pattern and the diffraction phase angle of the phyto-synthesized AgNPs are given in Figure 1 C. The diffraction peaks were observed at the angle of 38°, 44°, 64° and 77°, which are analogous to (111), (200), (220) and (311) Miller indices, respectively. The XRD analysis exhibit sharp peak patterns that indicate the presence of AgNPs in the sample. Our results are according to the previously published literature [24].

### 3.3. Agro-Morphological Parameter of Sunflower Plants Treated with AgNPs

**Figure 2:**
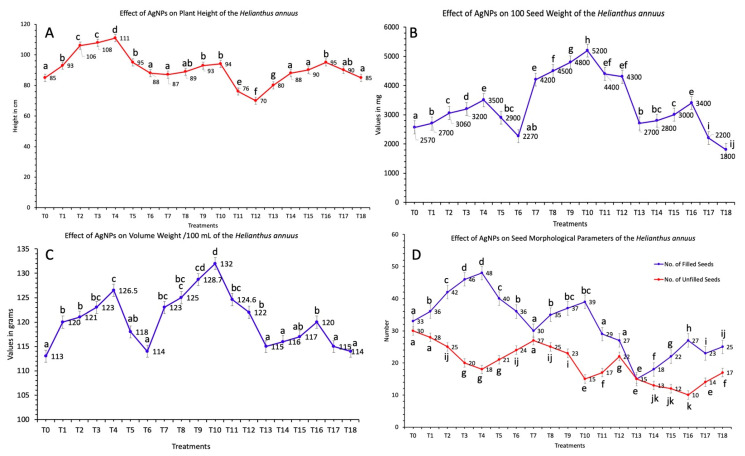
Effects of different treatments of AgNPs on (**A**) plant height, (**B**) 100 seed weight, (**C**) seed volume weight per 100 milliliters and (**D**) number of filled and unfilled seeds. Lowercase letters differed significantly at 5% probability levels, using Duncan’s Multiple Range Test (*p* < 0.05).

Greenhouse experiments were performed to assess the effects of AgNPs on sunflower plants’ morphological parameters. In the current study, the exogenous applications of AgNPs on the sunflower seeds increased the plant height to 111 cm when a 60 mg/L concentration of AgNPs was used to treat seeds. The minimum plant height (70 cm) was reported when 100 mg/L concentration of AgNPs was applied in combination of foliar sprays and seeds treatment while the height of the control plants without any treatment was reported at 85 cm (Figure 2A) (*p* < 0.05). It was reported that AgNPs alter the chemical nature of the plant growth media. The capillary action and the root pressure extracts excess nutrient from the soil through the process of sedimentation. In addition, a strong and extensive root branching system also enhances nutrient availability which helps to increase the plant height and other morphological parameters [1,2].

The head diameter reached its maximum (8.7 cm) at the concentration of 10 mg/L and the minimum size (3.2 cm) was reported at 100 mg/L concentration of AgNPs as compared to control (5.9 cm). The volume weight of seeds per 100 mL and 100 seed weight started increasing after the applications of AgNPs. The highest activity increase (132 g, seed volume weight per 100 mL and 5,200 mg, 100 seed weight) was reported at 60 mg/L of concentration when AgNPs were applied in combination of seed treatment and foliar sprays (Figure 2B,C). The shoot length or the plant height revealed a useful link with the head diameter of the flower, seed volume weight per 100 mL and 100 seed weight (*p* < 0.05). These results indicated that the higher concentration of bio-fabricated AgNPs was not effective for significant results. The applications of AgNPs beyond an optimal concentration pose toxic effects and resulted in the inhibition of the growth activity which was recorded by a decrease in plant agro-morphological parameters.

Interaction of the nanoparticles with the cellular physiological and molecular processes varies from species to species and depends on the size, shape, biochemical nature and concentration of nanoparticles [33,34,35]. The applications of the AgNPs helps in the efficient uptake of the nutrient for the improvement of organic nitrogenous compounds, photosystems, respiration, electron transport chain and oxidoreductive removal of reactive oxygen species. However, the higher concentration of AgNPs on sunflower plants has toxic effects which result in the reduction of agro-morphological and biochemical attributes [36,37].

A previous scientific investigation showed that the foliar spray of the low concentration of nano-zinc sulfide on sunflower plants increased the head diameter and the seed weight [38,39]. The head diameter of the flower is closely associated with the number, weight and size of the seeds. The number of the unfilled seeds showed a maximum (48) when 60 mg/L concentration of AgNPs were used to treat seeds and unfilled seed weight showed a maximum at 5,200 mg when 60 mg/L concentration of bio-fabricated AgNPs were used in combination of seeds treatment and foliar sprays on plants (Figure 2D). The highest number of unfilled seeds was calculated at 27 when a 10 mg/L concentration of AgNPs was used in combination of foliar sprays and seed treatment (*p* < 0.05). It was also reported that the number of the filled seeds reduced per capitulum in all treatments.

**Figure 3:**
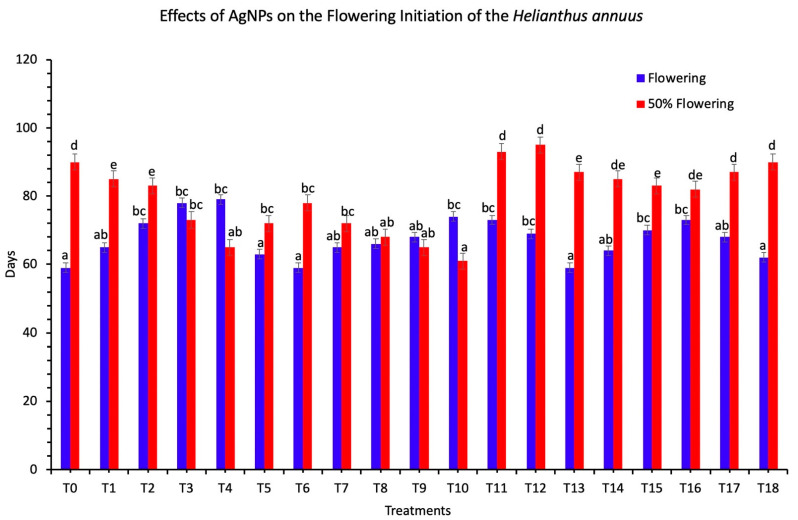
Effects of different treatments of AgNPs on sunflower flowering initiation. Lowercase letters differed significantly at 5% probability levels, using Duncan’s Multiple Range Test (*p* < 0.05).

In the current study, it was also examined that there was a reduction in the number of days of the flowering and 50% of flowering after the applications of AgNPs (Figure 3). The 50% of flowering was reduced to 61 days when 60 mg/L concentration of AgNPs were used in combination. The flowering was recorded at 59 days when 100 mg/L concentration of AgNPs was applied in the form of seed treatment (*p* < 0.05). These results show that the applications of AgNPs result in the reduction of the days to initiate flowering. Our findings were similar to those in Reference [40].

### 3.4. Antioxidant Enzymatic Potential of the Sunflower Plants Treated with AgNPs

The plant cells produce reactive oxygen species during the metabolic activity of NADPH oxidases which accumulate in different organelles of the plant cells, such as chloroplast, mitochondria, endoplasmic reticulum and plasma cell membrane [41,42]. These reactive oxygen species play an important role in signal transduction to produce enzymatic stress in the plant cell. The balance between the synthesis of ROS to maintain the cell defense and their neutralization is important to regulate the normal cellular physiological functions [43,44].

**Figure 4:**
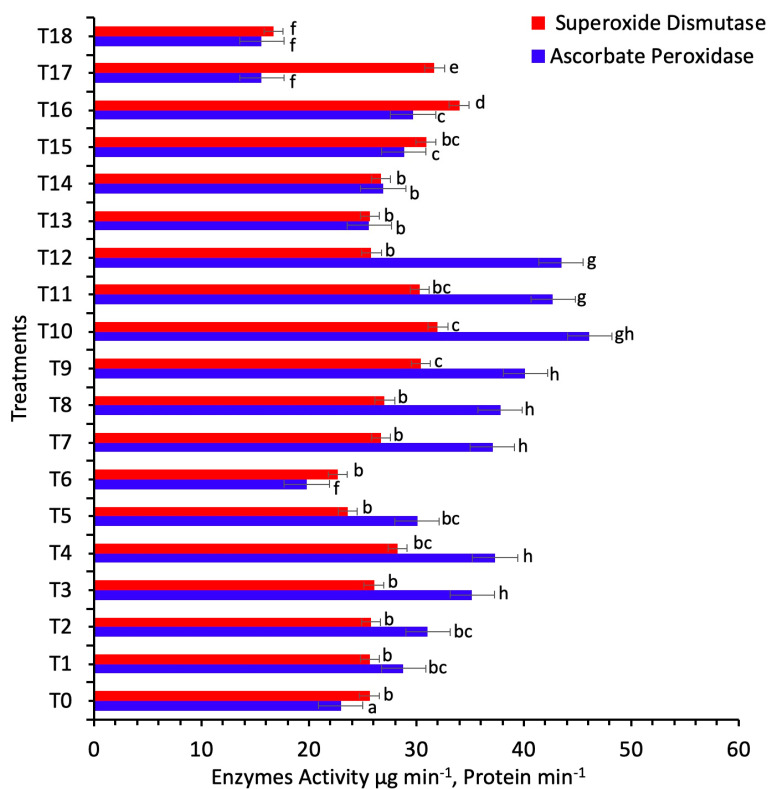
Effects of different treatments of AgNPs on superoxide dismutase and ascorbate peroxidase contents of sunflower. Lowercase letters differed significantly at 5% probability levels, using Duncan’s Multiple Range Test (*p* < 0.05).

In the present study, the enzymatic potential of the sunflower plant in response to various concentrations of AgNPs was determined in terms of SOD and peroxidase activities (Figure 4). The superoxide dismutase (SOD) increased their activity at 60 mg/L (foliar spray) concentration of AgNPs, which showed 34 mg/protein, and minimum activity was observed at 100 mg/L, which showed 17 mg/protein over the control (26 mg/protein) (*p* < 0.05). Maximum activity of ascorbate peroxidase (APx) was reported at 60 mg/L concentration of AgNPs, which showed 46 mg/protein (foliar spray and seed treatment), and minimum activity was observed at 100 mg/L (16 mg/protein, foliar spray) (*p* < 0.05). An elevated level of SOD and APx in comparison to the control showed the stress-protective response of the plant against the ROS created by AgNPs (*p* < 0.05). Ascorbate acts as an excellent reducing agent to convert the H_2_O_2_ to H_2_O, catalyzed by the enzyme ascorbate peroxidases [45]. Ascorbate peroxidase is localized in the plant cell, usually in chloroplast, mitochondria, peroxisome and cytosol [25]. The reactive oxygen species in the intracellular organelle can be controlled by the ROS-scavenging pathways. The ascorbate–glutathione cycle plays a significant role in the consecutive oxidation and reduction of ascorbate, glutathione and NADPH, catalyzed by the ascorbate peroxidase and superoxide dismutase [25]. Findings from the current study show that the ascorbate–glutathione cycle increased their activity at a concentration of 60 mg/L concentration of AgNPs when applied in combinations. Similar results have been reported by Reference [45] for rice plants, when the different concentrations of AgNPs increased the antioxidant activity to ameliorate the biotic stress.

### 3.5. Biochemical Parameter of the Sunflower Plants Treated with AgNPs

#### 3.5.1. Chlorophyll Contents

The biochemical attributes of the sunflower plants were analyzed to investigate the effects of the biosynthesized AgNPs. The determination of the contents of the photosynthetic pigments plays a significant role to determine the efficiency of plants to produce carbohydrate metabolites. Chlorophyll is an essential component of the chloroplast and is directly involved in carbohydrate metabolism during the process of photosynthesis. Photosynthesis plays a crucial role in plant growth and development, and the growth of plants improves by increasing the chlorophyll content [45,46,47].

**Figure 5:**
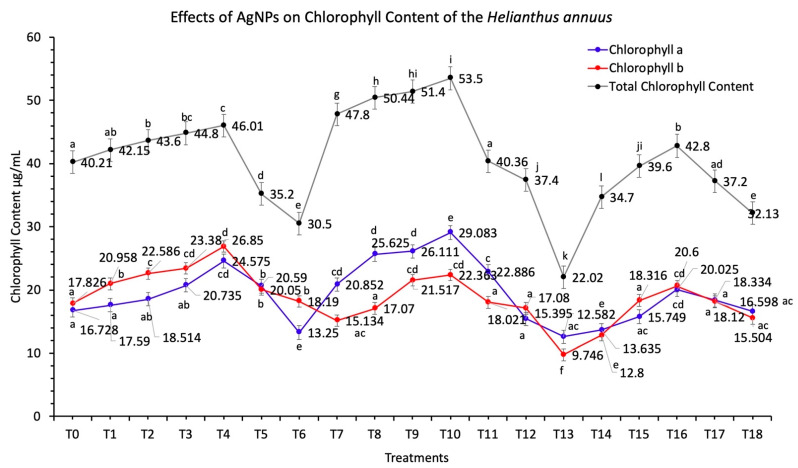
Effects of different treatments of AgNPs on chlorophyll contents of sunflower. Lowercase letters differed significantly at 5% probability levels, using Duncan’s Multiple Range Test (*p* < 0.05).

Applications of AgNPs improved the chlorophyll *a* and chlorophyll *b* contents (Figure 5). The maximum activity was reported at a 60 mg/L concentration of AgNPs when applied in combinations while the activity was reduced at 10 mg/L concentration of AgNPs (*p* < 0.05). Total chlorophyll contents increased at the 60 mg/L concentration of AgNPs, and the activity was recorded at 53.5 μg/mL. The minimum total chlorophyll content was recorded at 22.0 μg/mL, when a 10 mg/L concentration of AgNPs was applied in the form of foliar sprays in comparison to the control (40.21 μg/mL). Due to the increased concentration of AgNPs above the level of 60 mg/L, the AgNPs show a reduction in the activity of two important enzymes δ-aminolaevulinic acid dehydratase (al dehydratase) and protochlorophyllide, which ultimately result in to decline in the biosynthesis of chlorophyll [48,49].

Due to the presence of silver ions in the plant cells, the magnesium ions will not be properly adjusted in the tetrapyrrole ring of the chlorophyll [48]. A study has found that the AgNPs influence the light-harvesting protein complex II contents on the thylakoid membrane, which increases the energy transfer in photosystem II [49,50]. References [39,49] reported that the enhancement of hill reactions and chloroplast activity by TiO_2_ nanoparticles resulted in oxygen liberation and ferricyanide photoreduction.

The oxidative stress caused by the presence of AgNPs affects the photosynthetic apparatus, which in turn alters the physiological attributes of plants. Oxidative stress is also responsible for decreasing the photosynthesis rate by disruption of the thylakoid membrane and chlorophyll pigment in the plant cells [51,52,53].

#### 3.5.2. Proline Content

**Figure 6:**
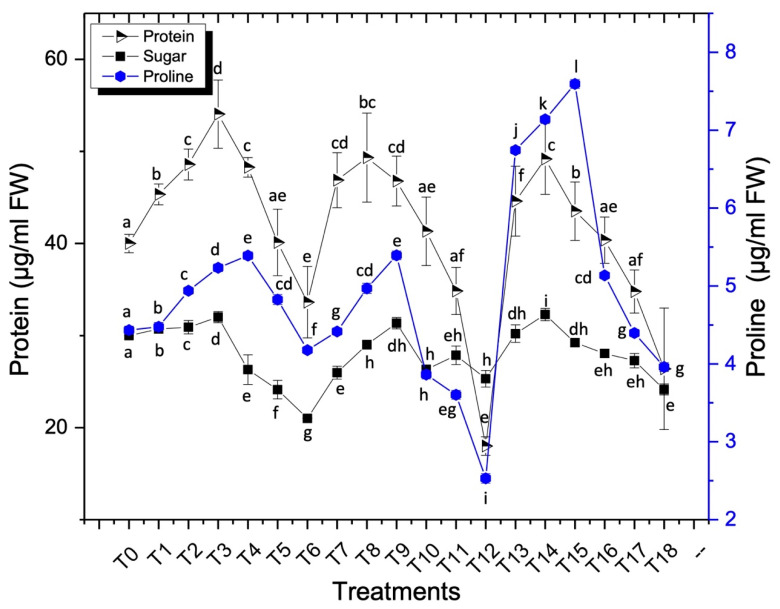
Effects of different treatments of AgNPs on protein, proline and soluble sugar contents of sunflower. Lowercase letters differed significantly at 5% probability levels, using Duncan’s Multiple Range Test (*p* < 0.05).

Proline presence plays an important role to normalize the stress conditions. Proline has a protective role to maintain an osmotic environment and redox balance. It also plays a significant role to protect the proteins from denaturation due to the stress caused by metallic nanoparticles thus maintains normal cellular physiological pathways [28,47]. A considerable increase in proline contents was observed in sunflower plants after the applications of AgNPs. Maximum accumulation of proline was recorded at 7.5 μg/mL FW when 40 mg/L concentration of AgNPs was used in the form of foliar sprays (Figure 6). The lowest proline activity was recorded at 100 mg/L concentration of AgNPs when applied in combination with foliar sprays and seed treatment, and the value was recorded at 2.5 μg/mL FW over control (4.4 μg/mL FW) (*p* < 0.05). Nitrogen in proline is the source of energy and plays a significant role as an osmoprotectant to maintain the stability of the intracellular organelle. It was reported earlier that there was the minimum loss of oxidative damage in the presence of proline under environmental stress [47].

#### 3.5.3. Soluble Sugar Contents

Soluble sugars play a promising role in plant defense mechanisms. According to the results, a considerable amount of the soluble sugars was accumulated in the plants after the applications of AgNPs, in comparison to the control plants (Figure 6). In the current study, the increased soluble sugar content was recorded at a 40 mg/L concentration of AgNPs when applied in combinations of seed treatment and foliar spray, while the value was recorded at 32 μg/mL. A minimum activity was recorded at 21 μg/mL, when 100 mg/L concentration of AgNPs was used in the form of seed treatment over control (30 μg/mL) (*p* < 0.05). It was reported earlier that the exposure of AgNPs increases the concentration of soluble sugar contents, which increases the reactive oxygen species, while the glutathione level goes down [54].

#### 3.5.4. Protein and Amino Acid Contents

Silver nanoparticles increased the protein activity at the concentration of 40 mg/L, when the seeds were treated and the values were recorded at 54 μg/mL FW, and the minimum value was recorded at 18 μg/mL FW when a 100 mg/L concentration of AgNPs was applied in combinations over control (40 μg/mL FW) treatment (*p* < 0.05) (Figure 6).

**Figure 7:**
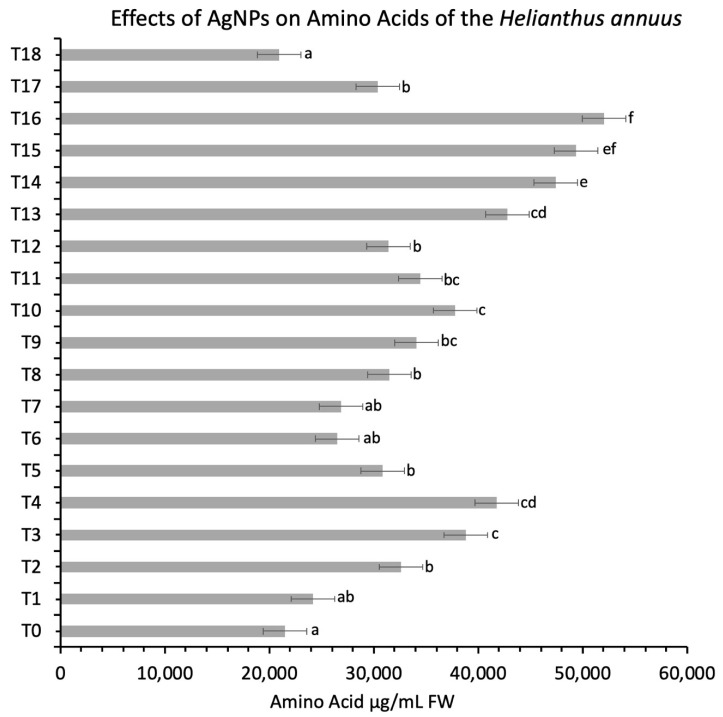
Effects of different treatments of AgNPs on amino acid contents of sunflower. Lowercase letters differed significantly at 5% probability levels, using Duncan’s Multiple Range Test (*p* < 0.05).

The amino acid has represented an increase in the activity of all treatments over control, which showed at 21,480 μg/mL FW (Figure 7) (*p* < 0.05). A similar result was reported when different concentrations (0, 30, 60 and 90 mg/L) of AgNPs were applied on *Pisum sativum* which reported a marked increase in protein and carbohydrate contents [55]. Increased protein content was also examined by applying the different concentrations of AgNPs on tomato plants [56]. It was also observed that increased protein quantity delimits the amino acid content by the application of AgNPs. The free amine group of protein attaches AgNPs; thus, stabilization by surface membrane protein is initiated [57]. Silver influences the proteins that are involved in the oxidation and reduction reaction, thus creating a harmful impact on homeostasis regulation [58].

### 3.6. Seed Quality Parameter and Fatty Acid Profiling of Sunflower Plants After the Applications of AgNPs

Sunflower oil is an important food source due to the presence of significant nutrient contents. A significant increase in the concentration of the linoleic acid was observed at 40 mg/L concentration of silver nanoparticles which was recorded at 58.58% and minimum values were recorded at 100 mg/L, which showed 56% over control (57%) (Table 2) (*p* < 0.05). The stearic acid contents decreased in all concentrations of silver nanoparticles over control (6.6%), while the contents of the palmitic acid showed a minute increase (4.05%) at a concentration of 20 mg/L of AgNPs and minimum values were recorded at 3% when 40 mg/L concentration of AgNPs were used over control (4%) (*p* < 0.05).

In the current research, the oil contents increased (0.66%) significantly at a 40 mg/L concentration of AgNPs and the minimum value was recorded at 0.46% over control (0.54%) when a 100 mg/L concentration of AgNPs were applied. Oleic acid contents were increased at a concentration of 80 mg/L of silver nanoparticles which showed a 29% increase and a minimum value was recorded at 27% when 100 mg/L concentration of AgNPs was used over control (28%) (*p* < 0.05). According to Yaseen et al. (2015), the use of AgNPs interferes with the orotic acid which reduces the quantity of saturated fatty acid and increases the quantity of unsaturated fatty acid (linoleic, α-linolenic and oleic acid). Oleic acid and linolenic acid are the bioactive metabolites of the sunflower plants that play their role in the synthesis of oil. A fatty acid is usually degraded by fatty acid hydrolases; therefore, a 30% to 50% reduction in the fatty acid synthesis and oilseed content was observed. Reference [13] reported that applications of AgNPs (25 mg/L and 50 mg/L) on sunflower increased linolenic acid content but the stearic acid content has been decreased as profiled in Table 1.

## 4. Conclusions

Food scarcity is a major challenge that the world is facing these days. The improvement of the plant yield and the quality can play a promising role to successfully tackle this growing need. In this study, we reported the green synthesis of silver nanoparticles by using the leaves’ aqueous extract of *Euphorbia helioscopia*. The morphological and optical analytical techniques confirmed the synthesis, and the nanoparticles were observed as being crystalline, having a size range of 30–100 nm. It was observed experimentally that the applications of silver nanoparticles on seeds and the foliar sprays have a positive impact on sunflower plants’ agro-morphological, antioxidant, enzymatic and oilseed and fatty acid contents. The applications of silver nanoparticles increased the flower head diameter, which was attributed to an increase in the seed weight and seed number. These findings conclude that the combinatorial applications of silver nanoparticles play a significant role to promote plant growth and development by altering the molecular and biochemical pathways. This experimental evidence provides a baseline study for the in-depth analysis to increase the secondary metabolites, oil contents and fatty acid profile of the sunflower at the industrial level and their commercialization.

## Figures and Tables

**Table 1:** Treatment layout of the study.

Treatment	Treatment Description	Concentration
T_0_	Control	0 mg/L
T_1_	Seed treated	10 mg/L
T_2_	Seed treated	20 mg/L
T_3_	Seed treated	40 mg/L
T_4_	Seed treated	60 mg/L
T_5_	Seed treated	80 mg/L
T_6_	Seed treated	100 mg/L
T_7_	Seed treated + Foliar spray	10 mg/L
T_8_	Seed treated + Foliar spray	20 mg/L
T_9_	Seed treated + Foliar spray	40 mg/L
T_10_	Seed treated + Foliar spray	60 mg/L
T_11_	Seed treated + Foliar spray	80 mg/L
T_12_	Seed treated + Foliar spray	100 mg/L
T_13_	Foliar spray	10 mg/L
T_14_	Foliar spray	20 mg/L
T_15_	Foliar spray	40 mg/L
T_16_	Foliar spray	60 mg/L
T_17_	Foliar spray	80 mg/L
T_18_	Foliar spray	100 mg/L

**Table 2:** Seed-quality parameters in terms of oilseed and fatty acid contents of sunflower.

Treatments	Seed Quality Parameter				
	Oil Content %	Palmitic Acid %	Stearic Acid %	Oleic Acid %	Linolenic Acid %
T_0_	0.54 d	6.6 a	4 b	28.56 e	58.53 c
T_1_	0.56 d	6.1 a	3.97 ab	28.9 e	58.53 c
T_2_	0.59 cd	5.93 b	3.82 ab	29.12 cd	59.23 d
T_3_	0.66 a	5.9 b	3 a	29.25 cd	59.8 d
T_4_	0.61 c	5.98 b	3.6 d	29 a	59.2 d
T_5_	0.57 cd	6.01 a	3.97 ab	28.7 e	58.86 c
T_6_	0.52 cd	6.38 a	4.2 b	28.43 e	58.37 c
T_7_	0.48 e	4.7 bc	3.73 cd	29.02 a	58.56 c
T_8_	0.54 cd	4.65 bc	3.7 cd	29.03 a	58.37 c
T_9_	0.58 cd	4.5 c	3.62 c	29.4 a	57.56 b
T_10_	0.59 cd	4.72 bc	3.75 cd	29.1 a	57.48 b
T_11_	0.56 d	4.82 bc	3.79 cd	29.8 cd	57.4 b
T_12_	0.5 d	4.97 bc	3.84 d	29.76 cd	57.3 b
T_13_	0.49 e	4.77 bc	3.8 d	29.53 cd	57.15 a
T_14_	0.54 d	4.71 bc	3.72 cd	29.01 a	56.94 ab
T_15_	0.58 cd	4.64 bc	3.67 c	28.72 e	56.74 a
T_16_	0.65 b	4.56 d	3.61 c	28.64 e	56.7 a
T_17_	0.61 c	4.67 bc	3.68 c	28.55 e	56.6 a
T_18_	0.57 cd	4.75 bc	3.73 cd	28.61 e	56.53 a

Lowercase letters differed significantly at 5% probability levels, using Duncan’s Multiple Range Test (*p* < 0.05).

## Data Availability

Complete data is available in the manuscript.
